# Evaluation of drug interactions in outpatients taking antipsychotic medications

**DOI:** 10.3389/fphar.2025.1590167

**Published:** 2025-05-14

**Authors:** Bshra A. Alsfouk, Qamar M. Aljanadi, Mona M. Almutairi

**Affiliations:** Department of Pharmaceutical Sciences, College of Pharmacy, Princess Nourah bint Abdulrahman University, Riyadh, Saudi Arabia

**Keywords:** antipsychotics, drug-drug interaction, drug-tobacco interactions, potential DDIs, psychiatric

## Abstract

**Introduction:**

Drug-drug interactions (DDIs) of antipsychotic medications are clinically significant as they can result in toxicity or treatment failure. This study aims to assess the potential drug-drug and drug-tobacco interactions associated with antipsychotic medications in an outpatient setting. Predictors of antipsychotic DDIs and the impact of potential DDIs on patients’ clinical outcomes were also evaluated in this study.

**Methodology:**

A cross-sectional study was conducted on outpatients in King Fahad Medical City in Riyadh, Saudi Arabia between 25 October 2020, and 26 November 2020, who received antipsychotic medications. Data were collected using medical record review. Potential DDIs were assessed using electronic Lexicomp^®^. The identified potential DDIs were categorized based on risk rating, severity, and reliability rating. Potential adverse effects from interactions were classified by mechanism into pharmacodynamic and pharmacokinetic.

**Results:**

The study included 220 patients who received 804 drug combinations (i.e., ≥2 drugs concomitantly administered) between antipsychotics and other concomitant drugs. The commonest concomitant classes were antidepressants (20%), anticonvulsants (18%) and cardiovascular agents (15%). The rate of potential DDIs was 71% (n = 574/804). Of the DDIs identified, 92% and 7% were rated C (require monitor therapy) and D (require modify regimen), respectively. In terms of severity level, the majority (n = 552, 96%) of interactions were considered moderate and only 9 interactions were categorized as major. The level of scientific evidence was classified as fair in 64% and as good in 36% of interactions. The majority (91%) involved pharmacodynamic interactions rather than pharmacokinetic mechanisms (9%). The most frequent potential adverse effects were increased sedation (36%), hyperglycemia (15%) and decreased blood pressure (14%). Receiving polypharmacy (i.e., ≥5 drugs concomitantly administered) was significantly associated with an increased probability of drug interaction occurrence (OR = 42, P = 0.0026). Uncontrolled disease state was slightly higher in patients with potential DDIs compared to those with no DDIs (24% vs. 22%, P = 1). Likewise, adverse drug effects were significantly more common in patients with potential DDIs (89% vs. 72%, P = 0.014). The rate of potential drug-tobacco interactions was 6% of patients.

**Conclusion:**

Potential DDIs of antipsychotic drugs were frequent (71%) and were associated with increased adverse effects. It is crucial for the clinicians to be aware of DDIs, monitor patients closely, and make the appropriate interventions. This emphasizes the importance of enhancing the knowledge about DDIs and the use of reliable AI machines, such as clinical decision support systems, to prevent medication errors.

## 1 Introduction

Antipsychotic medications are widely used in the treatment of schizophrenia, bipolar disorder, and other psychiatric conditions (Kennedy et al., 2013; Spina and De Leon, 2007). These agents are often used long term because these conditions are chronic or lifelong (Spina and De Leon, 2007). Furthermore, antipsychotics are commonly used in combination with other psychotropic medications, particularly antidepressants and antiseizure medications (Kennedy et al., 2013). Therefore, there is a high probability of drug interactions that can cause unpredictable and complicated outcomes (Spina and De Leon, 2007; Bleakley, 2012).

Drug interactions are associated with longer hospital stays and higher treatment costs, and they are a significant contributing factor to the occurrence of adverse drug events (Moura et al., 2009; Dumbreck et al., 2015).

Drug-drug interactions (DDIs) are defined as clinically significant changes in a drug’s effects due to concomitant administration of another drug (Hines et al., 2012). The detrimental consequences of a drug interaction can lead to increased adverse effects and toxic levels of a drug, or in other cases can lead to loss of drug effectiveness and an increased risk of treatment failure and relapse (Bleakley, 2012). Increased adverse drug effects can result in poor adherence to antipsychotic medications and poor treatment outcomes (Kennedy et al., 2013; Alsfouk et al., 2023).

Drug interactions can be categorized into pharmacokinetic and pharmacodynamic interactions. Pharmacokinetic drug interactions lead to alterations in the medication (and/or its metabolites) absorption, distribution, metabolism, or excretion. Whereas pharmacodynamic drug interactions result in changes in the response of the drug and occur at the site of action of the drug without altering drug concentrations (Kennedy et al., 2013; Hines et al., 2012). The effects of pharmacodynamic interactions at the receptor level might be antagonistic, synergistic, or additive (Wijesinghe, 2016).

The hepatic cytochrome P450 enzymes substantially metabolize the majority of antipsychotics. The CYP1A2, CYP2D6, and CYP3A4 isoenzymes are particularly significant for antipsychotic metabolism. Therefore, CYP-mediated DDIs are frequent among patients with psychiatric disorders. Concomitant administration of CYP inducers or inhibitors may result in clinically significant reduced therapeutic efficacy or drug toxicity, respectively (Kennedy et al., 2013; Wijesinghe, 2016). Toxicity of antipsychotic medication may result in serious adverse effects such as extrapyramidal symptoms (EPS), QT prolongation, serotonin syndrome, and seizures (Spina and De Leon, 2007). Many patients with psychiatric disorders smoke. Tobacco smoking induces hepatic drug-metabolizing enzymes such as CYP 1A2, and therefore reduces the plasma concentration of multiple antipsychotic drugs (Desai et al., 2001). However, few studies evaluated the drug interactions between tobacco smoking and antipsychotic medications.

Pharmacoepidemiologic studies demonstrated that potential DDIs in patients with psychiatric disorders are common, with up to 88% observed prevalence rates (Aburamadan et al., 2021; Ranković et al., 2024; Bole et al., 2023; Borges et al., 2017). However, the majority of these studies included inpatients. The association between the occurrence of potential DDIs and several demographic and drug-related factors was examined. Among different variables investigated, the number of administered medications was consistently found to be a significant predictor of potential DDIs in patients with psychiatric illness (Aburamadan et al., 2021; Ranković et al., 2024; Borges et al., 2017). Likewise, a study conducted in Saudi Arabia concluded that multiple prescriptions and older age are important factors of potential DDIs among psychiatric patients (AlRuthia et al., 2019). The former study by AlRuthia et al. (2019) focused on the role of pharmacists in preventing DDI, and the authors recommend conducting studies in the region that explore the prevalence rate of potential DDIs.

The therapeutic goal of antipsychotic treatment is to maximize the therapeutic benefits while minimizing the adverse effects and drug interactions. A better understanding of drug interactions of antipsychotic medications, close monitoring of patients, and individualizing drug regimens are fundamental for favorable therapeutic outcomes (Kennedy et al., 2013).

This study aims to estimate the prevalence, severity, and classification of potential drug–drug interactions (DDIs) involving antipsychotic medications in outpatients; identify predictors of these interactions; evaluate their association with clinical outcomes; and explore potential interactions between antipsychotics and tobacco smoking.

## 2 Methodology

### 2.1 Study design and patients

This cross-sectional study was carried out in the psychological care department’s outpatient clinics at King Fahad Medical City in Riyadh, Saudi Arabia. Data were gathered from the medical records between 25 October 2020, and 26 November 2020. Adults (≥18 years old) who had been taking antipsychotic medication(s) for a minimum of 4 weeks before data collection began were included in the study.

### 2.2 Data collection

Patient demographics, clinical characteristics, and information about antipsychotic medications were extracted from the patients’ medical record through the use of a pre-made data collection form. Patient demographics included age, gender, and smoking status. The information on antipsychotic drugs comprised the following: the list of antipsychotic drugs, their number, name, and dosage; adverse drug effects; and the indication of antipsychotic and the state of disease control. Information on other concurrent medications and comorbidities was also gathered.

DDIs were assessed using online Lexicomp^®^ (Lexicomp^®^, 2024). The information about the potential DDIs were gathered from Lexicomp^®^ between 5 Dec 2022, and 8 Jul 2023. Lexicomp^®^ Drug Interaction serves as a clinical decision support tool designed to detect potential interactions between different medications. Lexicomp^®^ stands out as a reliable, evidence-based information source on drugs and is recognized for its exceptional performance in screening for drug DDIs. Previous studies have evaluated the performance of Lexicomp^®^ Drug Interaction as a screening tool for detecting DDIs (Nusair et al., 2020; Smithburger et al., 2010; Aljadani and Aseeri, 2018). In this study, we utilized Lexicomp^®^ to identify potential drug interactions, given its reported specificity (80%–90%) and sensitivity (87%–100%) (Barrons, 2004; Kheshti et al., 2016; Roblek et al., 2015).

### 2.3 Outcomes definition

The study’s primary endpoint was the incidence of potential antipsychotic DDIs. In this study, potential DDIs were defined as possible drug interactions that could happen when two or more medications are taken concurrently, regardless of the actual harm (Hines et al., 2012; Aburamadan et al., 2021).

The secondary outcome of this study was predictors of potential drug interactions of antipsychotic medications. The investigated variables were age, gender, polypharmacy and comorbidity. Each patient’s primary psychiatric disorder, for which antipsychotic medication was prescribed, was identified. Comorbidities referred to additional conditions that the patient had alongside the primary psychiatric disorder. Polypharmacy was defined as the concomitant use of five medications or more, this definition was based on the literature (Halli-Tierney et al., 2019; Masnoon et al., 2017). This study also assessed the occurrence of potential DDIs and the following clinical outcomes: adverse drug effects and uncontrolled disease status. The adverse effects of medications were the ones that patients mentioned during their regular clinical appointments, which clinicians assessed and recorded in their medical files.

**FIGURE 1:**
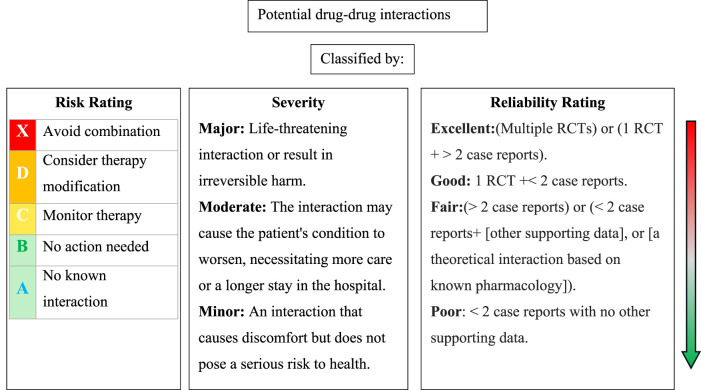
Classification of potential drug-drug interactions based on risk rating, severity, and reliability rating according to Lexicomp^®^ Drug Interaction. RCTs: randomized clinical trials.

As shown in Figure 1, each DDI was classified by risk rating, severity, and reliability rating according to Lexicomp^®^ Drug Interaction (Lexicomp^®^, 2024). The risk rating was categorized into five classes: X, D, C, B, and A. This classification serves as an indicator to aid in determining the appropriate response to interaction data. The transition from A to X signifies an escalating level of urgency in addressing the data. Broadly, A and B ratings suggest that the interactions have low clinical significance, whereas X, D, and C indicate situations likely to require a clinician’s attention. A detailed classification by risk ratings is demonstrated in Supplementary File S1. On the basis of level of severity, DDIs were classified into three categories: Major, Moderate, Minor, which are employed to determine the medical risk associated with the interaction. The reliability rating, also known as documentation or level of scientific evidence, reflects the quality and quantity of medical literature supporting the inclusion of data. It can be rated as Poor, Fair, Good, or Excellent.

Potential adverse effects from interactions between antipsychotics and concomitant medications were classified by mechanism into pharmacodynamic and pharmacokinetic. Pharmacodynamic adverse effects included increased sedation, hyperglycemia, decreased blood pressure or falls, QT-prolongation, seizure-potentiating effect, anticholinergic effect, increased weight gain, and effect reduction of concomitant medications (Bleakley, 2012). Pharmacokinetics involved inhibition or induction of cytochrome P450 3A4, 1A2, and 2D6 (Bleakley, 2012).

Concomitant drugs were prescribed drugs that were taken concurrently with antipsychotic medications by patients. USP^®^ Drug Classification 2023 (USP®DC. USP Drug Classification, 2023) was used to categorize concomitant drugs.

### 2.4 Statistical analysis

Numbers and percentages were used to represent categorical variables. Mean (±SD) was used to convey continuous variables. A multiple logistic regression test was performed to examine the association between potential drug interactions (dependent variable) and several demographic and clinical factors (independent variables). The investigated variables were age, gender, polypharmacy and comorbidity. A correlation test was conducted to assess the relationship between the number of prescribed medications and the number of potential DDIs. The chai-square (χ2) test was used to evaluate the impact of potential DDIs on patients’ clinical outcomes, including disease control state and adverse drug reaction reporting. A two-tailed p-value <0.05 was considered statistically significant. Data analysis was conducted using GraphPad Prism 10 (GraphPad Software, San Diego, CA, USA) and Microsoft Excel 16.77.1 statistical software.

### 2.5 Ethical consideration

This study was conducted in accordance with the ethical principles outlined in the Declaration of Helsinki and followed the guidelines of Good Clinical Practice (GCP). The King Fahad Medical City Research Ethics Committee, located in Riyadh, Saudi Arabia, granted the IRB approval for this study (IRB Number: 20-625), 22 September 2020. Throughout the study, patients’ privacy and confidentiality were strictly maintained.

## 3 Results

### 3.1 Demographic and clinical characteristics of patients

A total of 220 patients were included in the study. Table 1 summarizes the patients’ characteristics. The mean age of the patients was 42.1 years (SD = 15). The female gender was slightly predominant (52%). The most common psychiatric disorder that represented the primary indication for antipsychotic prescription was bipolar disorder (35%), followed by major depressive disorder (20%) and schizophrenia (19%). The psychiatric disorder was controlled in the majority of patients (76%). Around 67% of the patients had one or more other comorbidities. The most frequent comorbidities were anxiety disorders, depression, epilepsy, diabetes mellitus, hypertension, and dyslipidemia.

**TABLE 1:** Demographic and clinical characteristics of patients (n = 220).

Variables	Description	Count (%)
Age in years, Mean (SD), [range]		42. 1 (15), [18-80]
Gender	Female	115 (52)
	Male	105 (48)
Tobacco smoking	Yes	56 (25)
	No	164 (75)
Psychiatric disorder	Bipolar	76 (35)
	Depression	45 (20)
	Schizophrenia	41 (19)
	Psychosis	27 (12)
	Anxiety	14 (6)
	Others	17 (8)
Disorder control status	Controlled	167 (76)
	Uncontrolled	53 (24)
Presence of comorbidities	Yes	148 (67)
	No	72 (33)

### 3.2 Antipsychotic and concomitant drugs

All patients received antipsychotic medications; the majority (91%) took monotherapy of antipsychotic medications. The most commonly used antipsychotic was quetiapine (41%), followed by olanzapine (24%) and risperidone (22%). All prescribed antipsychotics were new-generation drugs, with the exception of two patients who received old-generation haloperidol.

A total of 96 patients (43.6%) were prescribed five or more medications, indicating polypharmacy. A total of 190 patients received at least one combination of antipsychotic and concomitant drugs, while the remaining 30 patients took a single antipsychotic with no concomitant drug. The combinations ranged from 1 to 21 per patient, with an overall total of 804 combinations in the study cohort. Approximately 46% of concomitant drugs were central nervous system (CNS) medications. The most frequently concomitant drug class was antidepressants (20%), followed by anticonvulsants (18%) and cardiovascular agents (15%). Table 2 shows the antipsychotic and concomitant medications received by the study patients.

**TABLE 2:** Prescribed antipsychotic and concomitant drugs to the patients.

Variables	Description	Count (%)
Antipsychotic medication regimens	Monotherapy	200 (91)
	Dual therapy	19 (8.5)
	Three antipsychotics	1 (0.5)
Antipsychotic medications	Quetiapine	98 (41)
	Olanzapine	57 (24)
	Risperidone	52 (22)
	Aripiprazole	32 (13)
	Haloperidol	2 (1)
Number of prescribed medications per patient, mean (SD)		4.8 (3.3)
Patients receiving polypharmacy (≥5 medications)		96 (43.6)
Patients receiving drug combination	Yes	190 (86.4)
	No	30 (13.6)
Total number of combinations between antipsychotic and concomitant drugs in the cohort		804
Concomitant drugs	CNS drugs	369 (46)
	Non-CNS drugs	435 (54)
Concomitant drug class	Antidepressants	160 (20)
	Anticonvulsants	145 (18)
	Cardiovascular Agents	124 (15)
	Blood Glucose Regulators	91 (11)
	Electrolytes/Minerals/Metals/Vitamins	67 (8)
	Gastrointestinal Agents	28 (3)
	Analgesics	27 (3)
	Hormonal Agents, Stimulant/Replacement/Modifying (Thyroid)	26 (3)
	Antipsychotics	21 (3)
	Anti-Parkinsonian Agents	21 (3)
	Others	94 (12)

CNS: Central Nervous System.

### 3.3 Potential drug-drug interactions (DDIs) between antipsychotic and concomitant drugs

Out of the 804 combinations, 574 (71%) had potential DDIs, the remaining 230 (29%) had no DDIs. The rate of potentially harmful (moderate and major) DDIs was 69.8% (n = 561/804).

**FIGURE 2:**
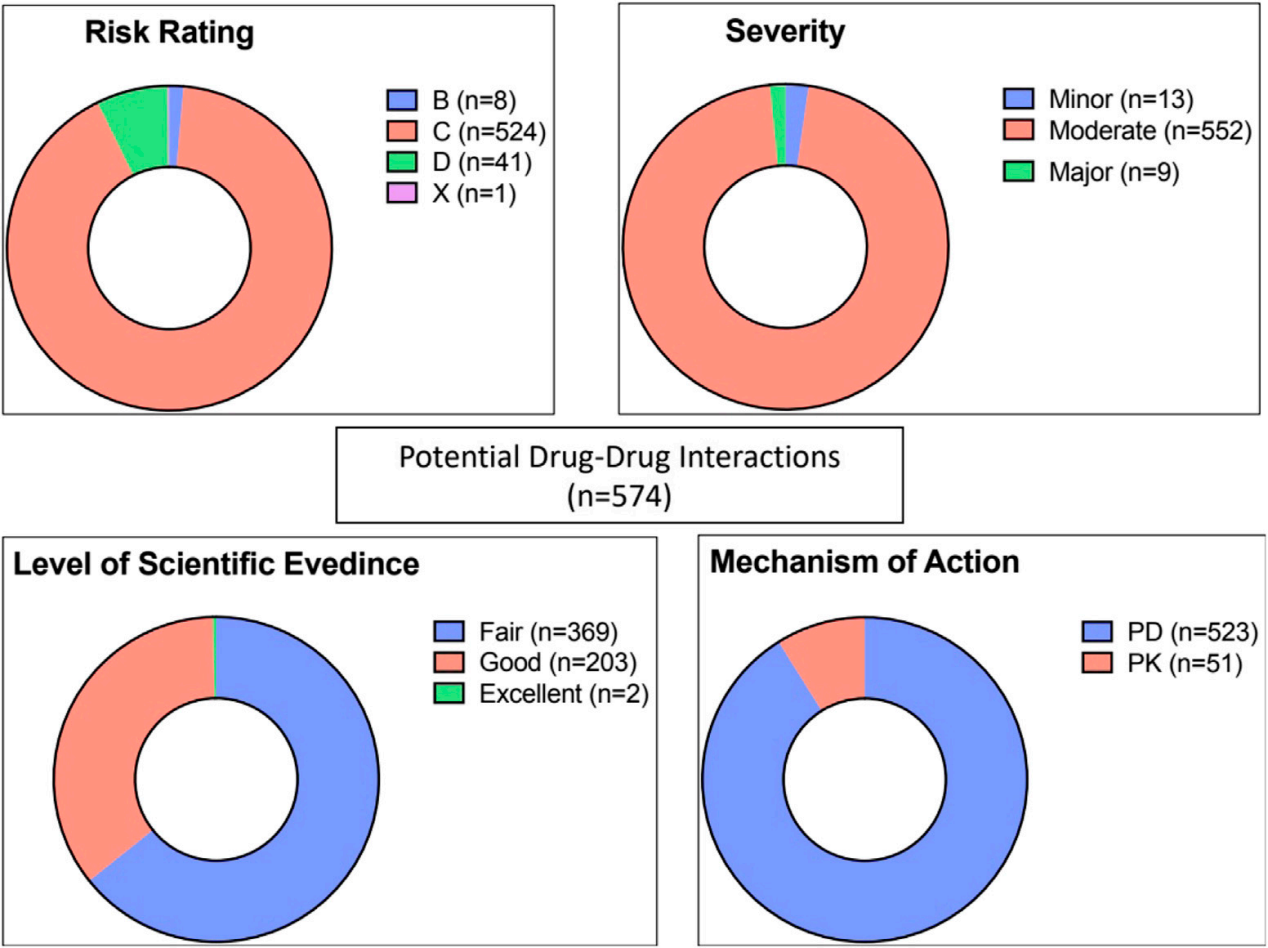
Risk rating, severity, reliability rating, and mechanism of potential drug-drug interactions (n = 574). PD: pharmacodynamic, PK: pharmacokinetic.

As demonstrated in Figure 2, of the 574 potential DDIs identified, 92% and 7% were rated C (require monitor therapy) and D (require modify regimen), respectively. There was one potential DDI with a risk rating of X (avoid combination), details of this interaction are shown in Table 3.

**TABLE 3:** Details of potential drug interactions with risk rating “X” or severity level of “Major”.

Combinations	Count	Description	Level of evidence
Potential drug interaction with a risk rating X			
Quetiapine- oral potassium chloride	1	Quetiapine has anticholinergic activity including decreasing gastrointestinal motility and secretion. Therefore, it may enhance the ulcerogenic effect of oral potassium chloride	Fair
Potential drug interactions with severity level of “Major”			
Aripiprazole-carbamazepine	2	Carbamazepine is a strong CYP3A4 inducer and decreases the serum concentration of concomitant aripiprazole and risperidone	Good
Risperidone-carbamazepine	2		Good
Aripiprazole-phenytoin	1	Phenytoin is a strong CYP3A4 inducer that decreases the serum concentration of aripiprazole	Good
Quetiapine-codeine	2	Antipsychotic medications potentiate the CNS depressant activity of codeine, an opioid agonist	Fair
Olanzapine-codeine	1		Fair
Risperidone-codeine	1		Fair

CNS: Central Nervous System.

In terms of severity level, the majority (n = 552, 96%) of interactions were considered moderate, and only 9 interactions were categorized as major (Table 3). The level of scientific evidence was classified as fair in 64% and as good in 36% of interactions. The majority (91%) involved pharmacodynamic interactions rather than pharmacokinetic mechanisms (9%).

As shown in Table 4, the most common potential adverse effects from interactions between antipsychotics and concomitant medications were increased sedation (36%), hyperglycemia (16%) and decreased blood pressure (14%). The details of potential drug interactions are explained in Supplementary File S1.

**TABLE 4:** Potential adverse effects from interactions between antipsychotics and concomitant medications (n = 574).

Potential adverse effects from interactions	Count (%)	Concomitant medications that interact with antipsychotics	Count
Increased sedation/toxicity	204 (36)	Anticonvulsants	88
		SSRIS/SNRIs	64
		Other antidepressants	16
		Benzodiazepines	14
		Baclofen/dantrolene	6
		Opioid Analgesics	5
		Others	11
Hyperglycemia*	89 (16)	Oral antidiabetic agents	59
		Insulins	30
Decreased blood pressure or falls	81 (14)	Beta-Adrenergic Blocking Agents	29
		Angiotensin-Converting Enzyme (ACE) Inhibitors	13
		Diuretics	13
		Calcium Channel Blocking Agents, Dihydropyridines	11
		Angiotensin II Receptor Antagonists	9
		Vasodilators	6
QT-prolongation	80 (14)	SSRIs/SNRIs/Tricyclics	57
		Antipsychotics	13
		Salmeterol/Salbutamol	5
		Antibiotics	4
		Dopamine antagonist	1
Seizure-potentiating effect	28 (5)	Antipsychotics	16
		Antidepressants (bupropion or tricyclics)	9
		Methylphenidate	3
3A4/1A2 induction	28 (5)	Carbamazepine (3A3/1A2 inducer)	19
		Phenytoin (3A3/1A2 inducer)	8
		Modafinil (3A4 inducer)	1
Anticholinergic effect	24 (4)	Procyclidine/Benztropine	15
		Trospium/Solifenacin	5
		Others	4
CYP inhibition	23 (4)	Fluoxetine/Paroxetine (2D6 inhibitors)	11
		Fluvoxamine (3A4/1A2 inhibitor)	7
		Others	5
Increased weight gain	10 (2)	Olanzapine + Valproate Sodium	10
Effect reduction#	6 (1)	Anti-Parkinson’s Agents	6
Ulcerative effect	1	Quetiapine-oral potassium chloride	1

SSRIs: Selective Serotonin Reuptake Inhibitors, SNRIs: Serotonin Norepinephrine Reuptake Inhibitors.

^*^Antipsychotics induce hyperglycemia that may reduce the therapeutic effect of glucose-lowering agents.

^#^Antipsychotics are dopamine antagonists that may reduce the therapeutic effect of anti-Parkinson’s agents, dopamine agonists.

### 3.4 Predictors of potential drug interactions of antipsychotic medications

To assess the association between the potential drug interactions and several demographic and clinical factors, a multiple logistic regression analysis was performed. The investigated factors were the patient’s age, gender, receiving polypharmacy, and presence of comorbidities. As demonstrated in Table 5, receiving polypharmacy was significantly associated with an increased probability of drug interaction occurrence. The odds of potential DDIs in patients who received polypharmacy were approximately 43 times that of patients who did not receive polypharmacy. Likewise, the presence of comorbidities was associated with an increased likelihood of potential DDIs but was not statistically significant. The remaining examined factors, age and gender, were not found to be associated with potential drug interactions.

**TABLE 5:** Multivariate logistic regression analysis for predictors of potential drug interactions with antipsychotic medications.

Factor	Adjusted OR (95% CI)	P – value
Age	0.9797 (0.9456–1.012)	0.2319
Gender	0.6100 (0.2249–1.637)	0.3243
Polypharmacy	42.52 (3.888–559.6)	0.0026*
Comorbidity	1.945 (0.7411–5.480)	0.1889

OR, Odds Ratio; CI, Confidence Interval.

Furthermore, the correlation test showed that the number of prescribed drugs had a significant positive correlation with the number of potential DDIs, Pearson r (95% CI) was 0.667 (0.5864–0.7344), P < 0.000.

### 3.5 Association between potential drug interactions and patients’ clinical outcomes

**FIGURE 3:**
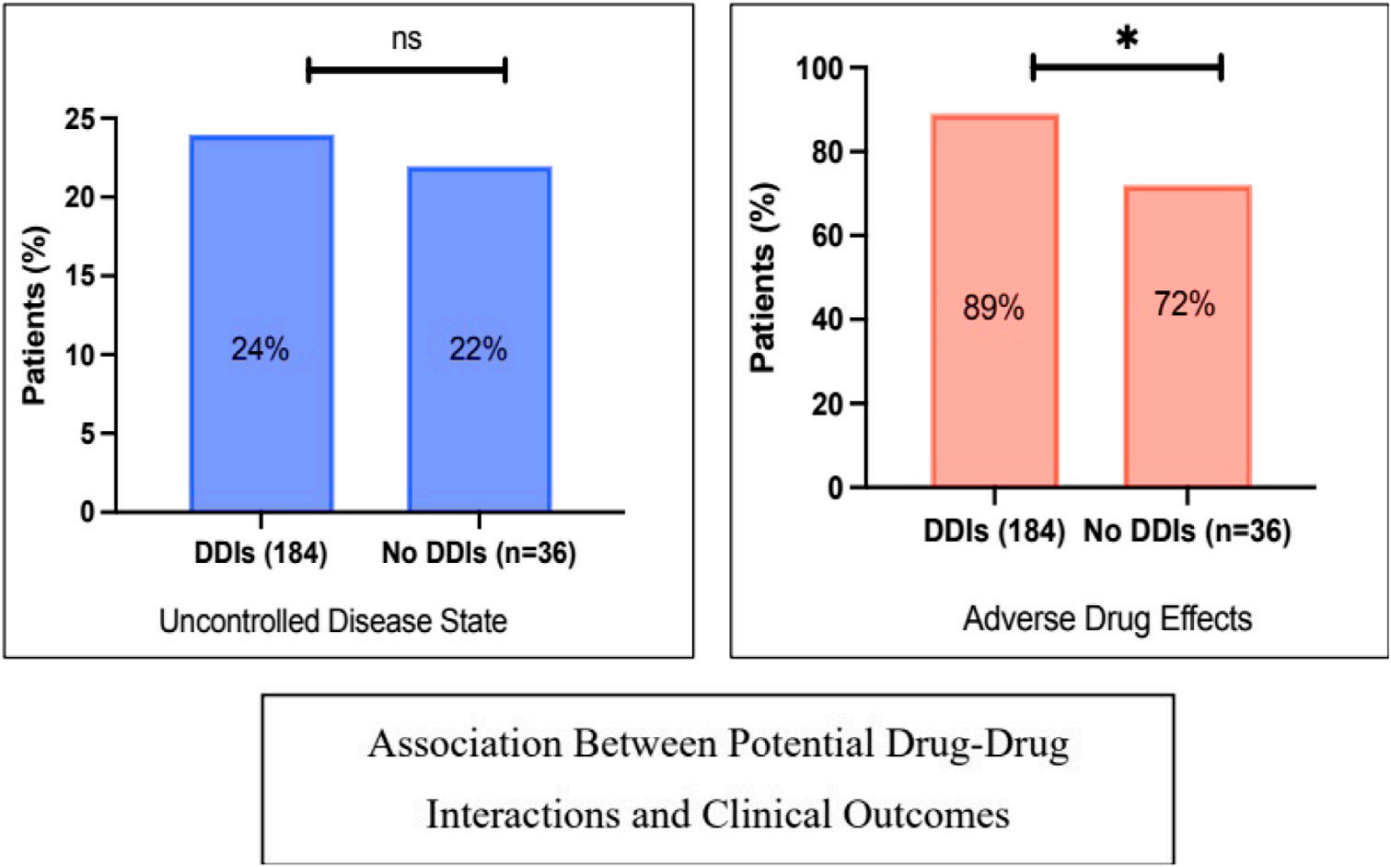
Associations between potential drug-drug interactions and patients’ clinical outcomes: rate of uncontrolled disease status and adverse effects. * denotes a significant p-value, ns: not significant. The X^2^ test was used.

The impact of potential DDIs on patients’ clinical outcomes was assessed. The investigated clinical outcomes were uncontrolled disease state and adverse drug reaction reporting. As illustrated in Figure 3, the rate of uncontrolled disease state was slightly higher in patients with potential DDIs compared to those with no DDIs (24% vs. 22%, P = 1). Likewise, adverse drug effects were significantly more common in patients with potential DDIs (89% vs. 72%, P = 0.014).

### 3.6 Smoking-antipsychotic interactions

Out of 220 patients, there were 14 (6%) cases of potential interactions between smoking and antipsychotic drugs: olanzapine (n = 13) and haloperidol (n = 1).

## 4 Discussion

Antipsychotic drug interactions are frequent and harmful. For positive therapeutic outcomes, it is essential to gain a deeper understanding of the pharmacological interactions of psychotropic medications, monitor patients closely, and customize drug regimens.

In the presented study, the most common concomitant drug class used in combination with antipsychotic medications was antidepressants which represented 20% of total combinations, followed by anticonvulsants (18%). The rate of concomitant combinations with CNS drugs was 46%. This result is in line with earlier research that demonstrated that concomitant use of antipsychotics, antidepressants, and benzodiazepines is frequent among patients with schizophrenia (Kennedy et al., 2013; Ocaña-Zurita et al., 2016; Mojtabai and Olfson, 2010; Guo et al., 2012; Acharya et al., 2023).

This study evaluated the prevalence rate, severity, and classification of potential DDIs of antipsychotic medications in outpatients. The findings of this study showed that the overall rate of potential DDIs was 71%, and the rate of harmful (moderate and severe) potential interactions was 69.8%, which are considered high incidence rates. The observed prevalence rate in this study

was in line with that reported by other studies, which ranged from 23% to 88% (Aburamadan et al., 2021; Bole et al., 2023; Borges et al., 2017; Guo et al., 2012). The lowest prevalence rate was reported by Guo et al. (2012), who conducted a population-based study on patients with schizophrenia and found that the rate of harmful (moderate and severe) potential drug interactions of antipsychotics was 23%. A study in the United Arab Emirates found that the overall frequency of potential DDIs of antipsychotic medications in inpatients was 64.7% (Aburamadan et al., 2021). Another study on inpatients with schizophrenia demonstrated that the prevalence of clinically relevant (type X and D) potential DDIs was 88% (Bole et al., 2023). However, the preceding two studies differ from the presented study in that they were on hospitalized patients. The study by Bole et al. (2023) reported a higher rate than that observed in the present study, this could be due to the nature of hospitalized patients, as they tend to have uncontrolled disorders that require more potent regimens of antipsychotics such as higher doses and combinations. Compared to our study, Bole et al. (2023) found a higher prescription frequency of combination therapy of antipsychotic medications, clozapine (which is reserved for pharmacoresistant cases), and old-generation antipsychotic drugs. In fact, few studies investigated potential DDIs of antipsychotic medication in outpatients. A study in primary healthcare demonstrated a prevalence of 58.4% (Borges et al., 2017). Another study, which included 492 outpatients, showed that half of the identified potential DDIs (47.6%) involved psychotropic medications (Marović et al., 2024). However, the studies by Borges et al. (2017), Marović et al. (2024) were on psychiatric patients who received any psychotropic medications, not only antipsychotic medications. The significant variation in reported prevalence rates of potential DDIs is likely influenced by differences in study design, population characteristics, healthcare settings, the drug interaction database used, and the criteria for defining potential DDIs, which complicates direct comparisons across studies. In general, it is consistently reported that drug interactions with antipsychotic medications are frequent and clinically relevant (Bleakley, 2012; Ocaña-Zurita et al., 2016; Spina et al., 2016).

This study demonstrated that pharmacodynamic interactions were far more common than pharmacokinetic, 91% and 9%, respectively. The most common potential complication due to pharmacodynamic interactions was increased sedation, which accounted for 36%. Other frequent and clinically relevant potential complications found in this study were hyperglycemia and decreased blood pressure, increased risk of QT-prolongation, and seizure-potentiation effects. This aligns with existing literature that demonstrates that the most frequent interactions occurring in clinical practice are pharmacodynamic interactions (Bole et al., 2023; Ocaña-Zurita et al., 2016; Guo et al., 2012; Collins et al., 2023). It is known that clinically significant pharmacodynamic interactions with antipsychotic medications may result in severe adverse drug effects including serotonin syndrome, QT prolongation, extrapyramidal symptoms, and seizure (Kennedy et al., 2013; Bleakley, 2012; Bačar Bole et al., 2023). Furthermore, a large study by Guédon-Moreau et al. (2004) investigated drug interaction in around five million prescriptions and demonstrated that antipsychotic medication class was one of the nine drug classes that account for most of the absolute contraindications, in which 54% were due to the combination of an antipsychotic (dopamine antagonist) with an anti-Parkinson’s drug (dopamine agonist), or due to QT prolongation risks. Therefore, it is crucial for the physicians and pharmacists to have good knowledge about DDIs and pharmacological effects of medications to prevent potential complications by adjusting drug doses and regimens.

This study evaluated the association between the occurrence of potential DDIs and several factors including patients’ age, gender, polypharmacy, and comorbidity. The only factor that this study found to be significantly associated with potential DDIs was polypharmacy. There was a significant correlation between the number of prescribed drugs and the number of potential DDIs. Many studies examined various demographic, pharmacologic, and clinical risk factors for DDIs. The number of prescribed medications was consistently observed as a predictor for DDIs (Aburamadan et al., 2021; Ranković et al., 2024; Borges et al., 2017; AlRuthia et al., 2019; Acharya et al., 2023). This emphasizes the importance of closely monitoring patients with polypharmacy for drug interactions. Other studies demonstrated that advanced age was a predictor for DDIs (Borges et al., 2017; AlRuthia et al., 2019; Kirilochev et al., 2019). Older patients are prone to drug interactions, possibly because comorbidities, polypharmacy, and diminished hepatic, renal, and cardiovascular function are common in aging.

This study evaluated the association between potential DDIs and two clinical therapeutic outcomes: treatment failure (i.e., uncontrolled disease state) and reported adverse drug effects by patients. The rate of uncontrolled disease state was slightly higher in patients with potential DDIs compared to those with no DDIs. Likewise, adverse drug effects were significantly more common in patients with potential DDIs. It is well recognized that antipsychotic drug interactions can affect the clinical outcomes and lead to loss of effectiveness and/or poor tolerability (Kennedy et al., 2013; Bleakley, 2012). Therefore, it is important to assess DDIs and the appropriateness of concomitant drugs in patients with poor drug response and/or poor tolerability.

The potential interactions between tobacco smoking and antipsychotic medications were investigated in this study. Tobacco smoking was common (25%) among the study patients.

It is important to note that patients on antipsychotic medication who smoke regularly can require larger dosages of antipsychotics than those who do not smoke (Sagud et al., 2009). This is due to a decrease in the anticipated plasma concentrations of several antipsychotics including olanzapine and clozapine, caused by the stimulation of CYP 1A2 activity (Lucas and Martin, 2013). On the other hand, tobacco smokers may need to reduce their antipsychotic dosage after quitting smoking (Sagud et al., 2009). In this study, there was a 6% incidence rate of potential interactions between smoking and antipsychotic drugs, and the majority was with olanzapine.

Potential DDIs occur when a pair of medications that are known to interact are taken concurrently, regardless of the actual harm (Hines et al., 2012; Aburamadan et al., 2021). However, awareness of these predicted interactions, informing the patients and close monitoring for any adverse drug effects are essential for preventing actual DDIs and optimizing the treatment outcomes. Interventions to avoid DDIs include adjusting the doses of antipsychotic medications or selecting a medication with lower interaction probability (Bleakley, 2012). Moreover, DDIs are frequently under-documented in clinical practice. A study in psychiatric clinics reported that 65% of DDIs were not documented (Collins et al., 2023). The use of AI-driven tools, such as clinical decision support systems, is recommended to help detect and manage these interactions effectively.

This study utilized a reliable database (i.e., Lexicomp) to evaluate drug-drug interactions between antipsychotic and concomitant prescribed medications and drug-smoking interactions. However, interactions with over-the-counter (OTC) medications, herbal remedies, and alcohol, which is illegal in Saudi Arabia, were not evaluated. Future studies are encouraged to investigate these areas. The period of this study was relatively short, due to practical constraints, which may limit the generalizability of the findings. Further recent and extended studies are recommended to reflect the emergence of new medications and the incorporation of updated drug interaction evidence.

This study demonstrated that the incidence of potentially harmful drug interactions was around 70% in outpatients receiving antipsychotics. This study identified the most frequent combinations, potential adverse effects, and patients at risk. Combinations between antipsychotics and other CNS medications, particularly antidepressants and anticonvulsants, were common. The most common potential adverse effects resulted from pharmacodynamic interactions and included increased sedation, hyperglycemia, and decreased blood pressure. Polypharmacy was found to be the strongest predictor of potential DDIs. Understanding these aspects of DDIs helps prescribers, pharmacists, and clinicians to predict and prevent adverse drug events through close monitoring and individualizing drug regimens. This study emphasizes the importance of enhancing the use of AI machines in clinical practice, such as clinical decision support systems, that help in detecting and preventing medication errors.

## Data Availability

The raw data supporting the conclusions of this article will be made available by the authors, without undue reservation.
