# Supplementary material for: Evaluation of drug interactions in outpatients taking antipsychotic medications
Source: Front Pharmacol. 2025 May 14;16:1590167. doi: 10.3389/fphar.2025.1590167 (PMC12116316; doi:10.3389/fphar.2025.1590167)

Appendix 1. Most frequent potential drug- drug interactions associated with antipsychotics

| Drug-drug interactions | Count | Risk rating | Severity | Level of evidence | Pharmacological consequence |
| --- | --- | --- | --- | --- | --- |
| Quetiapine+Escitalopram | 21 | C | Moderate | Fair | **QT-prolongation**  QT-prolonging Antipsychotic may enhance the serotonergic effect of QT-prolonging Antidepressants |
| Risperidone+Escitalopram | 17 | C | Moderate | Fair | **QT-prolongation**  QT-prolonging Antipsychotic may enhance the serotonergic effect of QT-prolonging Antidepressants  **Increased toxicity**  Serotonergic Agent may enhance the adverse/toxic effect of Antipsychotic Agents. Specifically, serotonergic agents may enhance dopamine blockade, possibly increasing the risk for neuroleptic malignant syndrome. Antipsychotic Agents may enhance the serotonergic effect of Serotonergic Agents. This could result in serotonin syndrome |
| Quetiapine+Metformin | 15 | C | Moderate | Fair | **Hyperglycemia**  Hyperglycemia-Associated Agents may diminish the therapeutic effect of Antidiabetic Agents |
| Quetiapine+Insulin | 15 | C | Moderate | Fair | **Hyperglycemia**  Hyperglycemia-Associated Agents may diminish the therapeutic effect of Antidiabetic Agents |
| Quetiapine+Valproic Acid | 14 | C | Moderate | Good | **Increased sedation**  CNS Depressants may enhance the adverse/toxic effect of other CNS Depressants |
| Quetiapine+Risperidone | 13 | C | Moderate | Fair | **QT-prolongation**  Both antipsychotics have risk for QT prologation |
| Quetiapine+Venlafaxine | 13 | C | Moderate | Fair | **Increased toxicity**  Serotonergic Agent may enhance the adverse/toxic effect of Antipsychotic Agents. Specifically, serotonergic agents may enhance dopamine blockade, possibly increasing the risk for neuroleptic malignant syndrome. Antipsychotic Agents may enhance the serotonergic effect of Serotonergic Agents. This could result in serotonin syndrome. |
| Olanzapine+Valproic Acide | 10 | C | Moderate | Good | **Increased weight gain**  Both agents have risk to increase body weight.  **Enzyme inhibition**  Valproate may decrease the serum concentration of olanzapine |
| Risperidone+ Valproic Acid | 10 | C | Moderate | Fair | **Increased toxicity**  Valproate Products may enhance the adverse/toxic effect of RisperiDONE. Generalized edema has developed |
| Olanzapine+Bisoprolol | 9 | C | Moderate | Fair | **Decreased blood pressure or falls**  Blood Pressure Lowering Agents may enhance the hypotensive effect of Antipsychotic Agents |
| Quetiapine+Mirtazapine | 9 | C | Moderate | Good | **Increased sedation**  The concomitant use of two or more drugs that have the potential to depress CNS function |
| Quetiapine+Lamotrigine | 9 | C | Moderate | Good | **CNS depression**  CNS Depressants may enhance the adverse/toxic effect of other CNS Depressants |
| Olanzapine+Metformin | 9 | C | Moderate | Fair | **Hyperglycemia**  Hyperglycemia-Associated Agents may diminish the therapeutic effect of Antidiabetic Agents |
| Quetiapine+Olanzapine | 9 | C | Moderate | Fair | **QT-prolongation**  QT-prolonging Antipsychotics may enhance the QTc-prolonging effect of OLANZapine. |
| Quetiapine+Fluvoxamine | 8 | C | Moderate | Fair | **Increased toxicity**  Serotonergic Agents (High Risk) may enhance the adverse/toxic effect of Antipsychotic Agents |
| Risperidone+Benztropine | 7 | C | Moderate | Good | **Anticholinergic effects**  Anticholinergic Agents may enhance the adverse/toxic effect of other Anticholinergic Agents |
| Olanzapine+Fluoxetine | 7 | C | Moderate | Fair | **Increased toxicity**  Serotonergic Agents (High Risk) may enhance the adverse/toxic effect of Antipsychotic Agents. Specifically, serotonergic agents may enhance dopamine blockade, possibly increasing the risk for neuroleptic malignant syndrome. Antipsychotic Agents may enhance the serotonergic effect of Serotonergic Agents (High Risk) |
| Quetiapine+Bisoprolol | 7 | C | Moderate | Fair | **Decreased blood pressure or falls**  Blood Pressure Lowering Agents may enhance the hypotensive effect of Antipsychotic Agents |
| Quetiapine+Vortioxetine | 6 | C | Moderate | Fair | **Increased toxicity**  Serotonergic Agents (High Risk) may enhance the adverse/toxic effect of Antipsychotic Agents |
| Aripiprazole+Lamotrigine | 6 | C | Moderate | Good | **CNS depression**  CNS Depressants may enhance the adverse/toxic effect of other CNS Depressants |
| Olanzapine+Mirtazapine | 6 | C | Moderate | Good | **CNS depression**  Increase CNS Depressants may enhance the adverse/toxic effect of other CNS Depressants |
| Quetiapine+Gliclazide | 6 | C | Moderate | Fair | **Hyperglycemia**  Hyperglycemia-Associated Agents may diminish the therapeutic effect of Antidiabetic Agents |
| Quetiapine+Levetiracetam | 5 | C | Moderate | Good | **CNS depression**  CNS Depressants may enhance the adverse/toxic effect of other CNS Depressants |
| Quetiapine+Paroxetine | 5 | C | Moderate | Fair | **QT-prolongation**  QT-prolonging Agents may enhance the QTc-prolonging effect of QT-prolonging Agents (Highest Risk)  **Increased toxicity**  Serotonergic Agents (High Risk) may enhance the adverse/toxic effect of Antipsychotic Agents. Specifically, serotonergic agents may enhance dopamine blockade, possibly increasing the risk for neuroleptic malignant syndrome. Antipsychotic Agents may enhance the serotonergic effect of Serotonergic Agents (High Risk) |
| Quetiapine+Aripiprazole | 5 | C | Moderate | Good | **Seizure-potentiating effect**  Agents With Seizure Threshold Lowering Potential may enhance the adverse/toxic effect of Antipsychotic Agents. Specifically, the risk of seizures may be increased |
| Quetiapine+Duloxetine | 5 | C | Moderate | Fair | **Increased toxicity**  Serotonergic Agents (High Risk) may enhance the adverse/toxic effect of Antipsychotic Agents. Specifically, serotonergic agents may enhance dopamine blockade, possibly increasing the risk for neuroleptic malignant syndrome. Antipsychotic Agents may enhance the serotonergic effect of Serotonergic Agents (High Risk). |
| Abbreviations: CNS: Central Nervous System | | | | | |

Detail about risk rating of drug interaction according to Lexicomp® Drug Interaction.


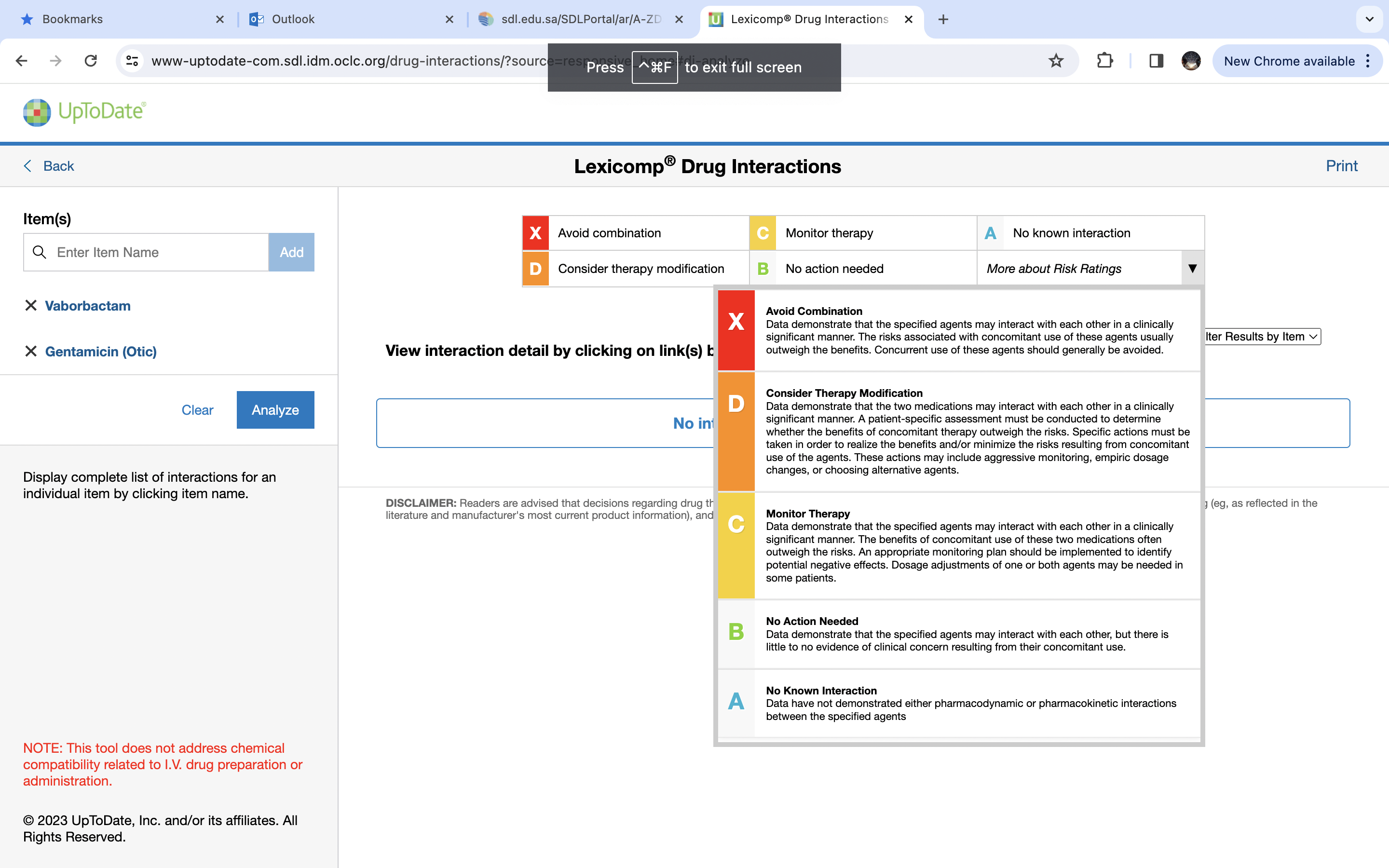

Supplement: Supplementary file 1 [file Supplementaryfile1.docx]
